# The NAMPT Inhibitor FK866 Increases Metformin Sensitivity in Pancreatic Cancer Cells

**DOI:** 10.3390/cancers14225597

**Published:** 2022-11-14

**Authors:** Maxime Parisotto, Nhung Vuong-Robillard, Paloma Kalegari, Thulaj Meharwade, Loick Joumier, Sebastian Igelmann, Véronique Bourdeau, Marie-Camille Rowell, Michael Pollak, Mohan Malleshaiah, Andréea Schmitzer, Gerardo Ferbeyre

**Affiliations:** 1 Department of Biochemistry and Molecular Medicine, Université de Montréal, Montréal, QC H3C 3J7, Canada; 2 Montreal Clinical Research Institute (IRCM), 110 Pine Avenue West, Montreal, QC H2W 1R7, Canada; 3 Montreal Cancer Institute, CR-CHUM, Université de Montréal, Montréal, QC H2X 0A9, Canada; 4 Division of Cancer Prevention, Department of Oncology, McGill University, Montréal, QC H3T 1E2, Canada; 5 Department of Chemistry, Université de Montréal, Montréal, QC H2V 0B3, Canada

**Keywords:** pancreatic cancer, metabolism, metformin, NAD, NAMPT

## Abstract

**Simple Summary:**

Use of the antidiabetic drug metformin as a single antitumor agent has been disappointing in clinics. We sought to explain why cancer cells adapt to metformin treatment and to develop more effective drug combinations. We found that the antitumor actions of metformin involved a reduction in the NAD+/NADH ratio, and that cells compensate by increasing NAD biosynthesis. Combining metformin with a low dose of the NAD biosynthesis inhibitor FK866 showed superior antitumor activity with undetectable toxicity; both in cell culture and in mice. Transcriptome analysis revealed that the combination triggered the expression of genes that mediate oxidative stress and cell death. In general, this work suggests that targeting mitochondria and NAD biosynthesis can lead to effective antitumor therapies.

**Abstract:**

Pancreatic cancer (pancreatic ductal adenocarcinoma: PDAC) is one of the most aggressive neoplastic diseases. Metformin use has been associated with reduced pancreatic cancer incidence and better survival in diabetics. Metformin has been shown to inhibit PDAC cells growth and survival, both in vitro and in vivo. However, clinical trials using metformin have failed to reduce pancreatic cancer progression in patients, raising important questions about molecular mechanisms that protect tumor cells from the antineoplastic activities of metformin. We confirmed that metformin acts through inhibition of mitochondrial complex I, decreasing the NAD^+^/NADH ratio, and that NAD^+^/NADH homeostasis determines metformin sensitivity in several cancer cell lines. Metabolites that can restore the NAD^+^/NADH ratio caused PDAC cells to be resistant to metformin. In addition, metformin treatment of PDAC cell lines induced a compensatory NAMPT expression, increasing the pool of cellular NAD^+^. The NAMPT inhibitor FK866 sensitized PDAC cells to the antiproliferative effects of metformin in vitro and decreased the cellular NAD^+^ pool. Intriguingly, FK866 combined with metformin increased survival in mice bearing KP4 cell line xenografts, but not in mice with PANC-1 cell line xenografts. Transcriptome analysis revealed that the drug combination reactivated genes in the p53 pathway and oxidative stress, providing new insights about the mechanisms leading to cancer cell death.

## 1. Introduction

With a 5-year survival rate of 10% and a median survival of 6 months after diagnosis, pancreatic cancer is one of the deadliest cancers [1]. Most patients have distant metastasis at diagnosis and therapies are often only palliative [2]. Moreover, the incidence of this cancer is expected to increase in the next years, while the therapeutic arsenal remains limited [3]. Metformin has been prescribed to diabetic patients for decades due to its very potent anti-hyperglycemic properties. Retrospective meta-analyses have suggested a protective effect of metformin against pancreatic cancer in type-II diabetes patients [4]. However, clinical trials using metformin as adjuvant treatment in pancreatic cancer did not show any beneficial effect [5,6]. Even if a slight effect may have been observed in low-grade tumor patients, metformin has no effect on high-grade tumors [7]. Despite the lack of conclusive results in patients, metformin has demonstrated potent anti-cancer properties in vitro and in preclinical in vivo experimental models [8,9,10,11,12,13,14]. These conflicting results raise questions about the response of cancer cells to metformin. We, more specifically, wondered whether cancer cells would exhibit intrinsic and/or treatment-induced resistance to metformin.

Several studies have shown that metformin targets mitochondria, thereby perturbing cellular energetics, via a mechanism relying on mitochondrial electron transport chain (ETC) complex-I inhibition [10,11,12,14]. ETC complex-I performs the oxidation of the reduced form of nicotinamide adenine dinucleotide (NADH) to regenerate the NAD^+^ needed as a cofactor for numerous catabolic reactions, including glycolysis, β-oxidation, and the Krebs cycle. Through inhibition of ETC complex-I, metformin was shown to decrease the cellular NAD^+^/NADH ratio, and manipulations to compensate or reverse this decrease reduced the sensitivity of cancer cells to metformin [12,15,16,17]. Herein, we show that PDAC cells treated with metformin increase NAD levels and glycolysis, but inhibition of the NAD salvage pathway with FK866 reverted this effect, sensitizing cells to cell death. Surprisingly, combining metformin and FK866 leads to reactivation of several p53 target genes, as well as genes that promote oxidative stress, suggesting new mechanisms which can be used to kill pancreatic cancer cells.

## 2. Materials and Methods

### 2.1. Cells and Reagents

Human PDAC cell lines PANC-1 (ATCC: CRL-1469), SW1990 (ATCC: CRL2172), HPAF-II (ATCC: CRL-1997), KP4 (Riken), and PSN1 (ATCC: CRM-CRL-3211) were cultured in Dulbecco’s modified eagle’s medium (DMEM) (Wisent, St-Bruno, QC) supplemented with 10% fetal bovine serum (FBS) (Wisent). Mouse cell lines 4T1 (ATCC CRL-2539) and MC38 (provided by Dr Pollak) were cultured as above. For in vitro assays, FK866 and metformin hydrochloride were purchased from Sigma-Aldrich. FK866 was suspended in DMSO and metformin in DMEM supplemented with 10% FBS. HPNE hTERT cells (ATCC) were cultured according to provider instructions.

### 2.2. Dose-Response & Growth Assays

Cells were seeded in 96-well plates. After 24 h, treatments were applied and cells were grown for three more days before fixation. Cell growth was assessed by crystal violet retention assays, which have been described previously [18]. The data are expressed as relative absorbance of crystal violet extracted from the cells and diluted in 10% acetic acid.

### 2.3. NAD/NADH Quantification

NAD/NADH quantitation colorimetric kit (#K337-100) from Biovision was used according to the manufacturer’s instructions.

### 2.4. DCFDA Measurements

For ROS measurements, cells were incubated for 30 min at 37 °C with 2 μM of dichlorodihydrofluorescein diacetate (DCFDA, Molecular Probes). Cells were collected using Trypsin, washed twice with PBS, and resuspended in 250 µL of FACS buffer (PBS, 1% BSA, 0.05 sodium azide). Measurements were made using BD FACS Symphony A3 and the acquired data were analyzed using FlowJO software.

### 2.5. Seahorse Analysis

KP4 cells were seeded in Seahorse XFe24 Fluxpak cartridge (Agilent) and treated with 5 nM FK866 or vehicle for 18 h. Then, media containing treatment were renewed before seahorse analysis. The ECAR (extracellular acidification rate) and OCR (oxygen consumption rate) of KP4 cells were measured on a Seahorse XF-24 (Agilent). Next, 10 mM Metformin or vehicle was added to cells after 30 min of seahorse measurement in order to establish baselines of OCR and ECAR.

### 2.6. Animal Experiments

All experiments were performed in accordance with the rules of the in vivo ethical committee of University of Montreal (CDEA #17-103) and CRCHUM (C18046GFs). For this study, 6–7-week old female nude mice (Hsd:Athymic Nude- Foxn1nu, Envigo) were implanted subcutaneously with 750,000 KP4 or 1,000,000 PANC-1 cells in 100 μL of 20% Matrigel (Corning) in saline. Tumor volume was determined by using a caliper, following the formula 4/3*π*(L*W*T), where L represents the length of the tumor, W the width, and T the thickness (all measured in millimeters). For in vivo experiments, metformin was purchased from Sigma-Aldrich (PHR1084) and FK866 from Selleckchem (S2799). The compounds were dissolved in the vehicle: 45% Propyleneglycol (Sigma-Aldrich) + 5% Tween 80 (Sigma-Aldrich) + ddH2O. Mice were injected intraperitoneally (with 100 μL) 5 days per week, starting on day 11 post-engraftment, with either 75 mg/kg/d metformin, 20 mg/kg/d FK866, metformin and FK866 combined (Met+FK; 75 mg/kg/d and 20 mg/kg/d, respectively), or vehicle for 4 weeks.

### 2.7. Tissue Histology

Tumors and organs collected at necropsy were rinsed in PBS, then weighed before formalin fixation and paraffin embedding. Tissue sections were cut at 4 microns, stained with hematoxylin and eosin (H&E), then scanned with the Aperio VERSA Brightfield Scanner. Tumor necrosis was unbiasedly quantified using Visiomorph software (Visiopharm, Hoersholm, Denmark), where a colour deconvolution algorithm specific to H&E was applied to the entire scanned tissue. Areas displaying low nuclear (hematoxylin) density are commonly considered to be necrotic tissue in pathology, so we applied the same principle for quantification: % necrotic area = areas of low nuclear density/whole tissue area. No differences in histology were observable by H&E or by the eye when assessing for organ toxicity, so quantification was not performed.

### 2.8. Total RNA Isolation and Sequencing

KP4 cells were seeded in 6 cm plates and treated with DMSO (vehicle), Metformin 10 mM, FK866 5 nM, or Metformin 10 mM and FK866 5 nM for 24 h. Cells were washed with PBS1X and collected in Trizol (#15596018, Invitrogen), and RNA extraction was performed using the RNeasy® Mini Kit (#74104, QIAGEN), according to the manufacturer’s instructions. cDNA libraries for sequencing were prepared using the Kapa RNA HyperPrep Kit with RiboErase (Roche) and UDI 96 indexes (Illumina). Sequencing was performed using NovaSeq 6000 flow cell S4 PE100.

### 2.9. RNA-Seq Data Analysis

RNAseq reads were trimmed using cutadapt (v3.7+galaxy0) with the following parameters: -a AGATCGGAAGAGCACACGTCTGAACTCCAGTCAC -A AGATCGGAAGAGCGTCGTGTAGGGAAAGAGTGT; trim, minimum-length 20, quality-cutoff 20. Reads were aligned to the human reference genome (hg38) using RNA STAR (v2.7.8+galaxy0). Gene quantification was performed using feature counts (Galaxy Version 2.0.1+galaxy2) with parameters -p -B. The gene counts were normalized using DEseq2 and subsequently used for principal component analysis (PCA) and detection of differentially expressed genes (DEGs). Genes with adjusted *p*-values < 0.05 and fold change > 1.5 were considered as significant DEGs. The analysis was performed using Galaxy (Galaxy version 22.05.rc1). Gene Set Enrichment Analysis (GSEA version 4.2.3) was used for functional annotation. The significance of enrichment values (NES) was determined by the False Discovery Rate (FDR; q-value) and by a nominal *p*-value [19]. Raw RNAseq data are available at GSE210562.

### 2.10. qPCR

Cells were collected as described before, and RNA extraction was performed according to the manufacturer’s instructions. RT-qPCR was performed as published in [20]. qPCR primers and their sequences are available in Supplementary Table S1.

### 2.11. Immunoblots

For immunoblots, adherent cells and floating cells were washed twice with PBS1X and lysed in 500 mL of Laemmli 2X Buffer (4% SDS, 20% Glycerol, 0.125 M Tris-HCl pH = 6.8). Cells were boiled at 95 °C for 5 min and protein concentration was estimated from A280 using NanoDrop (ThermoFisher). Samples were prepared and diluted using Laemmli 2X Buffer, 10% 2-mercapthoethanol, and 0.05% bromophenol blue. Then, 25 µg of proteins were loaded per sample and migrated on an SDS-PAGE (BioRad). They were transferred onto 0.45 µm nitrocellulose membranes (BioRad) and blocked for 1 h in 5% milk diluted in TBS-T (TBS1X, 0.05% Tween20). After 3 washes for 10 min with TBS-T, membranes were incubated with the corresponding primary antibodies (in PBS1X, 0.1% BSA, 0.02% Sodium azide). For anti phospho-p53 (Ser15) (9284, dilution 1:1000, Cell Signaling) and anti p53 (DO-1/sc-126, dilution 1:1000, Santa Cruz) antibodies, membranes were incubated overnight at 4 °C. Anti-histone H3 (ab1791, dilution 1:15,000, Abcam) and anti β-Actin (8H10D10/3700, dilution 1:2000, Cell Signaling) were incubated for 30 min at RT. After primary incubation, membranes were washed 3 times for 10 min with TBS-T, and were then incubated with HRP-conjugated secondary antibodies (1:3000) in milk TBS-T for 1 h (phospho-p53 and p53 antibodies) or 30 min (anti-histone H3 and β-Actin antibodies). For secondary antibodies, we used goat anti-mouse IgG (H+L)-HRP (170-6516, dilution 1:3000, BioRad) and goat anti-rabbit IgG (H+L)-HRP (170-6515, dilution 1:3000, BioRad). Finally, after 3 washes for 10 min, they were incubated with ECL Western blotting reagent (RPN2106, Amersham), and a signal was acquired using Super-RX X-ray films (Fujifilm). For the stripping solution between incubation with different antibodies, we used a stripping buffer (25 mM glycine, 1% SDS in dH_2_O, pH 2.0).

## 3. Results

### 3.1. The NAD+/NADH Ratio Determines Metformin Sensitivity

**Figure 1:**
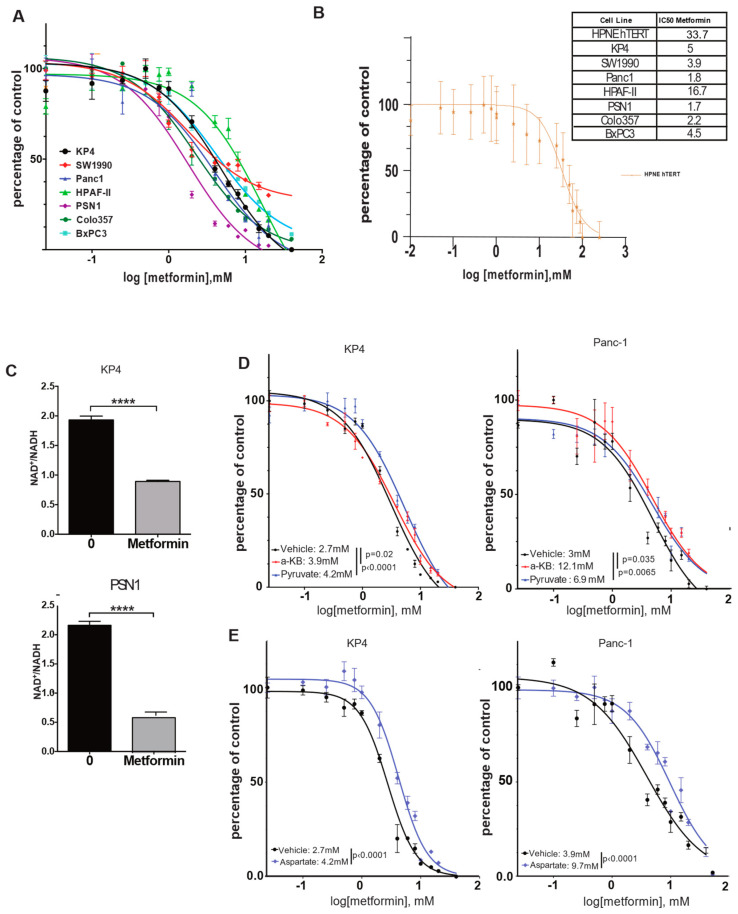
Metformin decreases growth of PDAC cells through a reduction in NAD^+^/NADH intracellular ratio in vitro. (**A**,**B**) Dose-response curves and IC50s of metformin on a panel of human PDAC cells (**A**) or HPNE cells (**B**) over 3 days of growth. Values are means of triplicates ± SEM. (**C**) Quantification of NAD^+^/NADH ratio in KP4 and PSN1 PDAC cell lines treated with 10 mM metformin or vehicle for 6 h. Values are means of triplicates ± SEM, **** *p*-value ≤ 0.0001 (Student’s *t*-test). (**D**,**E**) Dose-response curves and IC50s of metformin when PANC-1 and KP4 cells are on 4 mM α-ketobutyrate (α-KB), 1 mM of pyruvate (**D**), or 20 mM of aspartate (**E**) over 3 days of growth. Values are means of triplicates ± SEM. For all experiments, n = 3.

In order to determine the relative sensitivity to metformin of pancreatic cancer cell lines, we performed growth assays over 3 days in the presence of increasing concentrations of metformin on a panel of 7 human pancreatic cell lines and one non-tumorigenic immortalized human pancreatic ductal cell line, HPNE hTERT (Figure 1A,B). Note that all growth assays were performed in pyruvate-free medium, as pyruvate decreases the sensitivity of cell lines to metformin in vitro [15]. The growth of all pancreatic cancer cell lines was inhibited by metformin, with IC50s in the 1–10 mM range (Figure 1B). One notable exception was HPAF-II cells, which are slightly less sensitive to metformin, with an IC50 of ~16 mM. In contrast to cancer cell lines, the growth of non-tumorigenic HPNE hTERT cells was the least affected (IC50 33.7 mM). Such an important difference in metformin sensitivity between pancreatic tumor cells and non-tumorigenic cells was expected, and has already been reported [21]. With metformin being an inhibitor of mitochondrial ETC complex-I [12,15], these results are in agreement with the differential sensitivity of cancer cells versus normal cells to inhibitors of mitochondria ETC, as it appears that transformation of pancreatic cells is associated with an increase in mitochondrial mass/metabolism [13,22,23].

In order to better characterize the mechanism of growth inhibition of pancreatic cancer cells by metformin, we focused on its capacity to inhibit mitochondrial ETC complex-I. The inhibition of complex-I NADH dehydrogenase activity results in an increase in NADH level in cells, thereby decreasing the NAD^+^/NADH ratio [12,15]. Quantification of this ratio in KP4 and PSN1 cells after 6 h treatment with 10 mM metformin showed a reduction in NAD^+^/NADH by 50% and 70%, respectively, compared to vehicle-treated controls (*p*-values < 0.0001) (Figure 1C). This suggests that metformin acts in pancreatic cancer cells via mitochondrial complex-I inhibition.

It was shown previously that the NAD^+^/NADH ratio can be manipulated, to some extent, in vitro by the exogenous addition of lactate dehydrogenase A (LDHA) substrates such as pyruvate or α-ketobutyrate (α-KB), which act as alternative electron acceptors to re-oxidize NADH, thereby supplying NAD^+^ to the cells to run catabolic reactions [15]. In order to determine whether the decrease in the NAD^+^/NADH ratio by metformin contributes to its growth inhibition properties in pancreatic cancer cells, we repeated metformin IC50 measurements in KP4 and PANC-1 cells, either in the presence of pyruvate or α-KB or not. We observed that both compounds desensitized cells to the effect of metformin. For KP4, the IC50 was increased from 2.7 mM to 3.9 mM with α-KB (*p* = 0.02), and to 4.2 mM with pyruvate (*p* < 0.0001), while in PANC-1 cells, the IC50 was increased from 3 mM to 12.1 mM (*p* = 0.035) and 6.9 mM (*p* = 0.0065) with α-KB and pyruvate, respectively (Figure 1D). These results also reveal an important difference in the capability of pyruvate and α-KB to confer resistance to metformin depending on the cell line.

Upon ETC inhibition, cancer cell growth is highly dependent on aspartate synthesis [15,16,24]. Malate–aspartate shuttle is reversed to allow aspartate synthesis from malate to sustain cell proliferation, but this process requires NAD^+^. Hence, the lack of aspartate can limit growth after ETC inhibition. In agreement, the addition of 20 mM of aspartate to the culture medium of KP4 and PANC-1 cells increased their IC50 for metformin (Figure 1E) from 2.7 mM to 4.2 mM (*p* < 0.0001) and from 3.9 mM to 9.7 mM (*p* < 0.0001), respectively. Therefore, metformin decreases the growth of pancreatic cancer cells, at least in part, through inhibition of mitochondrial ETC complex-I and subsequent reduction in the NAD^+^/NADH ratio.

### 3.2. The NAMPT Inhibitor FK866 Increases Metformin Sensitivity

**Figure 2:**
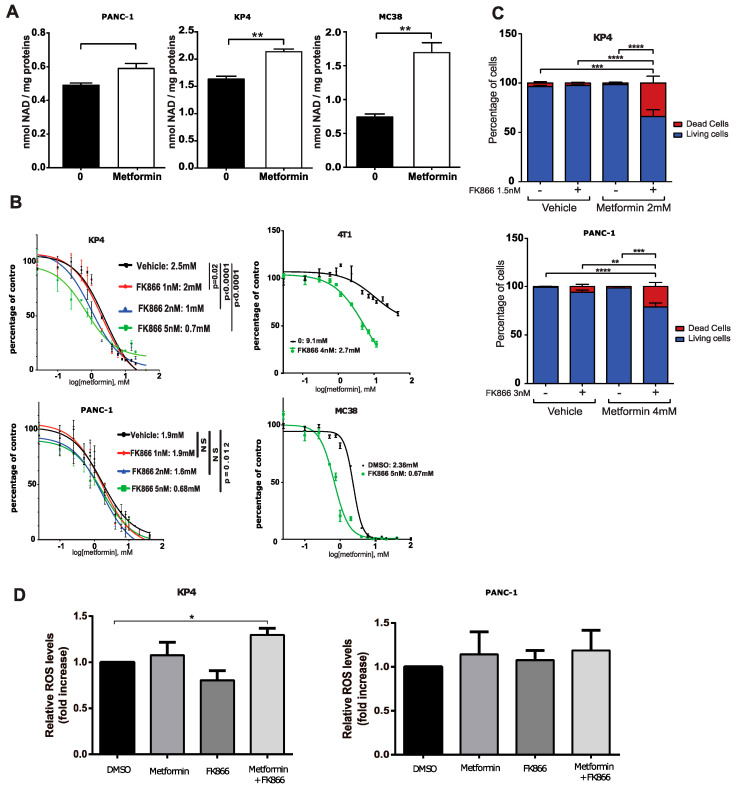
The NAMPT inhibitor FK866 and metformin cooperate to inhibit PDAC cells growth by strongly perturbing NAD metabolism. (**A**) Total NAD level in PDAC cells grown for 24 h in the presence or absence of 10 mM metformin. * *p*-value ≤ 0.05, ** *p*-value ≤ 0.01 (student *t*-test). (**B**) Dose-response curves and IC50s of metformin on growth of PDAC cell KP4 and PANC-1, breast cancer cells 4T1, and colon cancer cells MC38 treated with FK866 or vehicle for 3 days. (**C**) Viability (cell counts with trypan blue staining) of KP4 and PANC-1 cells treated with metformin or vehicle and FK866 or vehicle for 6 days. ** *p*-value ≤ 0.01, **** *p*-value ≤ 0.0001 (Student’s *t*-test). (**D**) Fluorescence intensity of KP4 and PANC-1 cells stained with DCFDA and measured by flow cytometry. Data show a relative change in median fluorescence intensity over control cells. Treatment for 24 h in the presence or absence of 10 mM metformin, 5 nM of FK866, a combination of metformin and FK866, or with vehicle. (**A**–**C**) Values are means of triplicates ± SEM, n = 3; for (**D**), values are means of duplicates ± SEM. ** *p*-value ≤ 0.01, *** *p*-value ≤ 0.001, **** *p*-value ≤ 0.0001 (ANOVA).

In an attempt to identify putative mechanisms of metformin resistance in cancer cells, we examined the NAD metabolism more closely. In addition to oscillating between two redox states in cells (namely NAD^+^ and NADH) in order to fulfill its high-energy electron carrier function, NAD is also the substrate for sirtuins (mono-ADP-ribosyltransferase or deacylase activity), Poly (ADP-ribose) polymerases (PARPs), and NADases, such as CD38. NAD^+^ is consumed by these enzymes, releasing nicotinamide (NAM) as a product. As a consequence, NAD^+^ needs to be resynthesized constantly in order to ensure the stability of the cellular pool [25]. We hypothesized that any metabolic process that can provide the cells with more NAD^+^, namely NAD synthesis pathways, would decrease sensitivity to complex-I inhibition by metformin. Indeed, we observed that upon 24 h of metformin treatment at 10 mM, total cellular NAD was increased by ~20% in PANC-1 and KP4 cells (*p* < 0.05 and *p* < 0.01, respectively) (Figure 2A). This increase is also markedly observed in the murine colorectal cancer cell line MC38 (Figure 2A), showing that this is a more general response to metformin and not a pancreas-specific trait. This result suggests that cancer cells may adapt to the effects of metformin by increasing NAD^+^ synthesis and/or decreasing NAD^+^ degradation.

Among these different metabolic pathways, the NAD salvage pathway has recently become an attractive target for anti-cancer therapies [26]. The NAD salvage pathway recycles NAM through its condensation with 5-phosphoribosyl-1-pyrophosphate (PRPP) to produce nicotinamide mononucleotide (NMN). This rate-limiting step of the salvage pathway is catalyzed by the enzyme nicotinamide phosphoribosyl transferase (NAMPT). NMN is then converted into NAD by the enzyme nicotinamide nucleotide adenylyl transferase 1 (NMNAT). In pancreatic cancer cells, the salvage pathway appears to be the predominant NAD synthesis pathway (as opposed to the De Novo synthesis pathway), partly because the enzyme NAMPT is overexpressed in human pancreatic cancers compared to normal pancreatic tissue [27,28].

In line with our hypothesis that enhanced NAD^+^ level could decrease metformin sensitivity, we treated KP4 cells and PANC-1 cells with increasing concentrations of the specific pharmacologic inhibitor of NAMPT, FK866 [29] to determine whether inhibition of this enzyme would increase metformin sensitivity. Indeed, FK866 sensitized KP4 and PANC-1 cells to the growth-inhibitory properties of metformin in vitro. FK866, at 5 nMm decreased the IC50 of metformin from 2.5 mM (vehicle) to 0.7 mM (*p* < 0.0001), and from 1.9 mM (vehicle) to 0.68 mM (*p* = 0.012) in KP4 and PANC-1 cells, respectively (Figure 2B). In order to determine if this cooperation is specific to PDAC cells or whether it also occurs in additional cancer types, we repeated the combinatorial treatment in the murine breast cancer cell line 4T1 and the murine colon cancer cell line MC38. FK866 decreased the IC50 of metformin from 9.1 mM (vehicle) to 2.7 mM (*p* < 0.0001), and from 2.36 mM (vehicle) to 0.67 mM (*p* < 0.0001) in 4T1 and MC38 cells, respectively (Figure 2C). Hence, NAMPT provided NAD+ and conferred metformin resistance across different cancer types.

In agreement with the sensitizing effect of FK866, the combination of metformin and FK866 (Met+FK) significantly increased cell death over 6 days of treatment in KP4 (~35%) and PANC-1 (~20%) cells compared to vehicle or to each compound alone (1–5%) (Figure 2D). Notably, a higher concentration of FK866 was necessary to inhibit PANC-1 cell growth with metformin and to induce cell death (Figure 2B,D). When used at similar concentrations, (Met+FK) only increased ROS in KP4 cells (Figure 2E). Together, these results show that metformin and the NAMPT inhibitor cooperate to inhibit growth and to induce oxidative stress and cell death of pancreatic cancer cell lines in vitro.

### 3.3. FK866 Inhibits Compensatory Glycolysis in Metformin-Treated Cells

**Figure 3:**
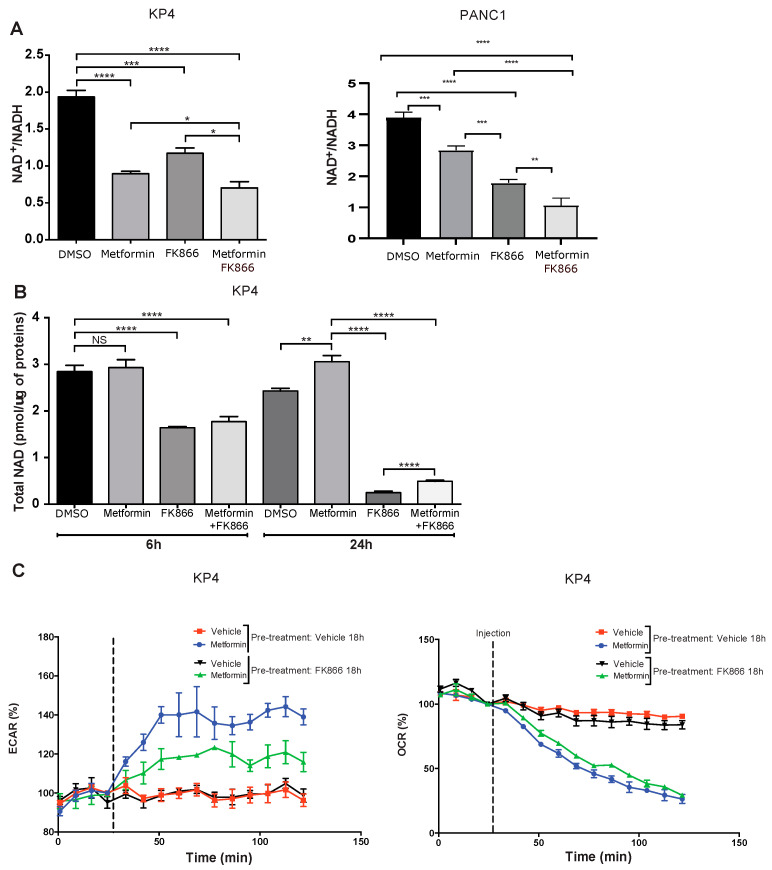
FK866 cooperates with metformin to reduce metabolic compensation. (**A**) Quantification of NAD+/NADH ratio in KP4 and PANC-1 cells treated for 6 h with 10 mM metformin, 5 nM FK866, a combination of metformin and FK866, or vehicle. (**B**) Quantification of total NAD levels after 6 h or 24 h in KP4 cells treated with 10 mM metformin, 5 nM FK866, Met+FK866, or vehicle. Values are means of triplicates ± SEM. * *p*-value ≤ 0.05, ** *p*-value ≤ 0.01, *** *p*-value ≤ 0.001, **** *p*-value ≤ 0.0001, NS, not significant (ANOVA), n = 3. (**C**) ECAR (extracellular acidification rate) and OCR (oxygen consumption rate) of KP4 cells treated with 10 mM metformin, measured by SeaHorse analysis. Cells were either pre-treated or not with FK866 (5 nM) for 18 h prior to SeaHorse analysis in order to deplete NAD levels. Values are means of triplicates ± SEM, n = 3.

Mechanistically, we observed in KP4 cells that both metformin (10 mM) and FK866 (5 nM) alone for 24 h decreased the NAD+/NADH ratio by ~50% and ~40%, respectively, compared to vehicle-treated cells (*p* < 0.0001 for metformin and *p* < 0.001 for FK866), while Met+FK further decreased the NAD^+^/NADH ratio (~60%) (Figure 3A). The effect of Met+FK on the ratio, although significant (*p* < 0.05), is relatively modest compared to either compound alone, and cannot account for the marked higher efficiency of the combination. In PANC-1 cells, the ratio of NAD^+^/NADH was higher, but it was also reduced by metformin and FK866. In addition to the decreased NAD^+^/NADH ratio, FK866 decreased the total pool of cellular NAD (NAD^+^ + NADH) after 6 and 24 h of treatment (Figure 3B). After 6 h, the decrease was ~50% compared to vehicle treated cells, whether in presence of metformin or not (*p* < 0.0001). After 24 h, FK866-treated cells exhibited an almost total depletion of NAD (~90%), while metformin slightly increased NAD levels, suggesting a tendency to replenish NAD in metformin-treated cells (Figure 3B).

Upon ETC inhibition by metformin, cancer cells switch to a glycolysis-based metabolism to generate ATP [22,30,31]. However, to sustain glycolysis, cells need to maintain a high NAD^+^/NADH ratio. Our results imply that cancer cells also need to sustain a high level of total NAD to provide enough cofactor for NAD-dependent glycolysis, independently of the ratio of NAD^+^/NADH. Indeed, it has been reported that inhibition of NAMPT decreases NAD levels and attenuates glycolysis in different cancer types, including pancreatic cancer cells [32,33,34,35]. Therefore, it is likely that the efficiency of the Met+FK combination in cancer cells is due to inhibition of the compensatory glycolytic flux, leading to a metabolic collapse. In agreement with this finding, treatment with FK866 reduced the extracellular acidification rate (ECAR) in metformin-treated cells, a measurement of compensatory glycolysis (Figure 3C). Notably, oxygen consumption rate (OCR) measurements show that FK866 did not further inhibit respiration in metformin-treated cells (Figure 3C). As normal cells are neither affected by metformin nor by FK866 due to a low expression of NAMPT [27], the effect of the Met+FK combination on cancer cells is expected to be relatively specific.

### 3.4. FK866 Improves Metformin Action on KP4 Xenografts in Nude Mice

**Figure 4:**
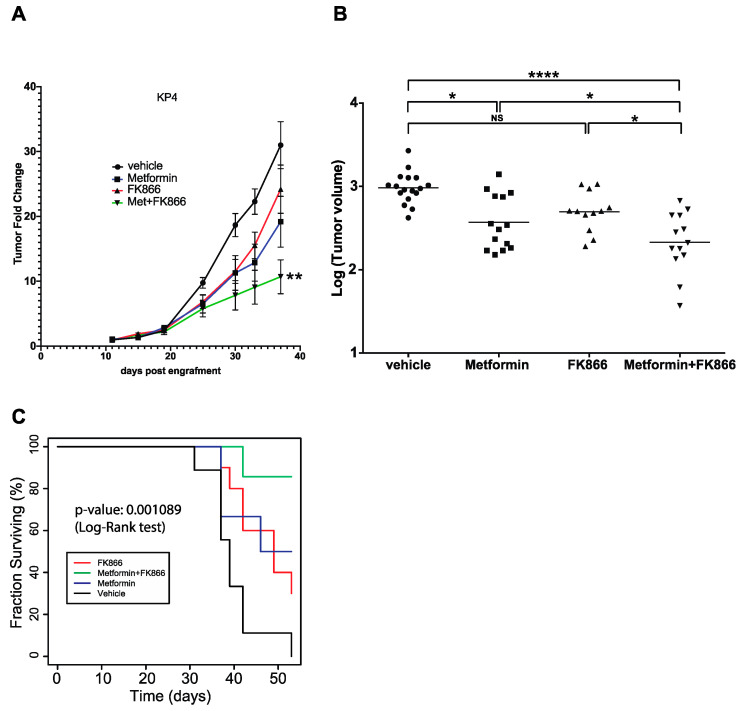
Progression of KP4 sub-cutaneous xenografts in nude mice. (**A**) Xenograft tumor growth of KP4 cells engrafted subcutaneously in nude mice. Metformin (75 mg/kg/d), FK866 (20 mg/kg/d), Met+FK, or vehicle treatments (intraperitoneally 5 days per week) were started 11 days post-engraftment. * *p* ≤ 0.01, ANOVA. (**B**) Graph showing the volume of each tumor after 37 days of the different treatments. * *p* ≤ 0.05, **** *p* ≤ 0.0001, NS: not significant (ANOVA). (**C**) Kaplan–Meyer survival curve of KP4 tumor-bearing mice as in (**A**) over 54 days.

In order to test the efficacy of the combination of FK866 and metformin in vivo, we performed xenograft experiments by initiating tumor formation of KP4 cells in nude mice. When the tumors (two per mouse, one on each flank, subcutaneously) reached ~100 mm^3^, the mice were randomly separated into four groups, receiving either vehicle, metformin alone (75 mg/kg/d), FK866 alone (20 mg/kg/d), or a combination of Met+FK intraperitoneally 5 times per week for 4 weeks. Met+FK showed a higher capacity to slow down KP4 tumor progression than either compound alone (Figure 4A). After 4 weeks of treatment (37 days after engraftment), the volume of the tumors in the Met+FK group was ~75% smaller than those in the vehicle group (*p* < 0.0001), and 40% smaller than the tumors in the metformin or FK866 groups (*p* < 0.05) (Figure 4B). In addition, the combination of Met+FK significantly improved survival relative to animals treated with either agent alone (Figure 4C, *p* = 0.001089). These results demonstrate that the combination is more potent for KP4 xenografts than either compound alone in vivo.

### 3.5. Metformin Does Not Cooperate with FK866 to Inhibit Growth of PANC-1 Xenografts in Nude Mice

**Figure 5:**
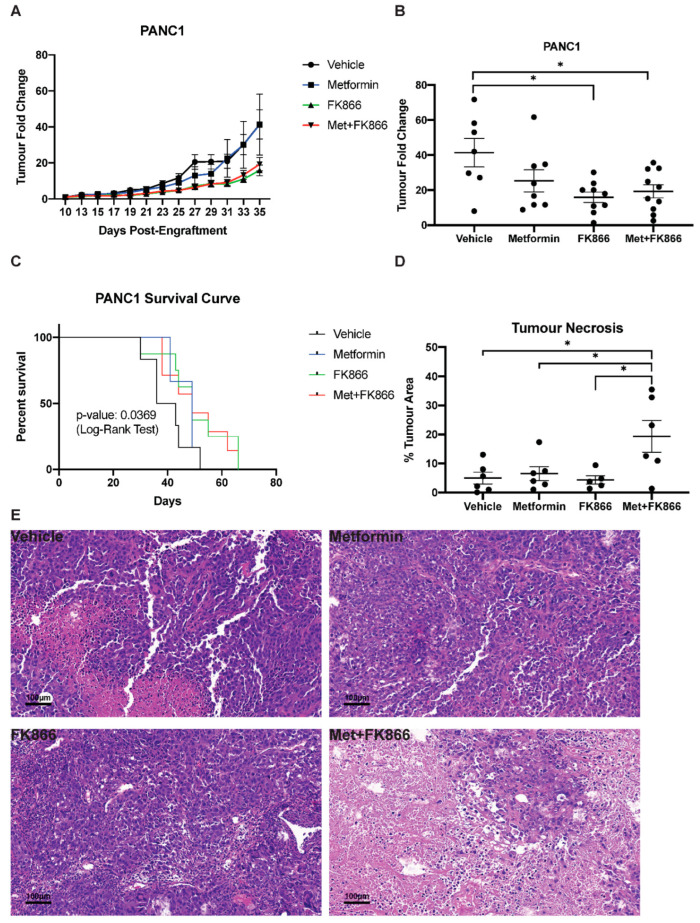
Progression of PANC-1 sub-cutaneous xenografts in nude mice. (**A**) Tumor fold change in mice bearing PANC-1 xenografts treated with Metformin, FK866, Met+FK, or vehicle (5 days a week). Treatment started 11 days post engraftment. (**B**) Tumor volume at day 35 from the mice represented in (**A**). (**C**) Kaplan–Meyer survival curves of PANC-1 tumor-bearing mice receiving different treatments over 65 days. (D-E) Quantification of % tumor necrosis (**D**) by H&E staining (**E**) of PANC-1 tumors, as in (**B**). (**B**–**D**) * *p* ≤ 0.05 (ANOVA).

Next, we tested the combination of Met+FK on PANC-1 tumors in nude mice. As before, the mice were randomly separated into four groups, receiving either vehicle, metformin alone (75 mg/kg/d), FK866 alone (20 mg/kg/d), or a combination of Met+FK. In order to match the treatment time frame of the KP4 xenografts, drug injections were started 11 days post-engraftment. In this setting, treatment with FK866 reduced tumor growth, but it was not further improved by metformin (Figure 5A,B). Similarly, FK866 improved survival relative to metformin and untreated mice, but the combination did not further improve survival (Figure 5C). Intriguingly, combining Met+FK triggered massive necrosis (Figure 5D,E). However, this necrosis does not explain the effect of the treatment, since FK866 alone had similar effects as Met+FK without it. Necrosis is a less efficient cell death mechanism, and, in some contexts, it can promote tumorigenesis [36]. It is then plausible that this necrosis, induced by Met+FK on PANC-1 tumors, compromised the efficacy of the combination in nude mice. Alternatively, our in vitro data show that PANC-1 cells require higher concentrations of metformin and FK866 to elicit cell death compared to KP4 cells (Figure 2D). Given that a phase 2 clinical trial has flagged potential toxicity with FK866 use [37], three mice were randomly selected from each treatment group after 4 weeks of drug treatment to assess for toxicity by gross examination and histological analysis. The dosage and schedule which we used did not reveal any major toxicity in the animals (Supplemental Figure S1), suggesting that the treatment for PANC-1 xenografts could be further optimized using higher doses.

### 3.6. Combining FK866 with Metformin Restores p53 Signaling in Cancer Cells with Mutant p53

**Figure 6:**
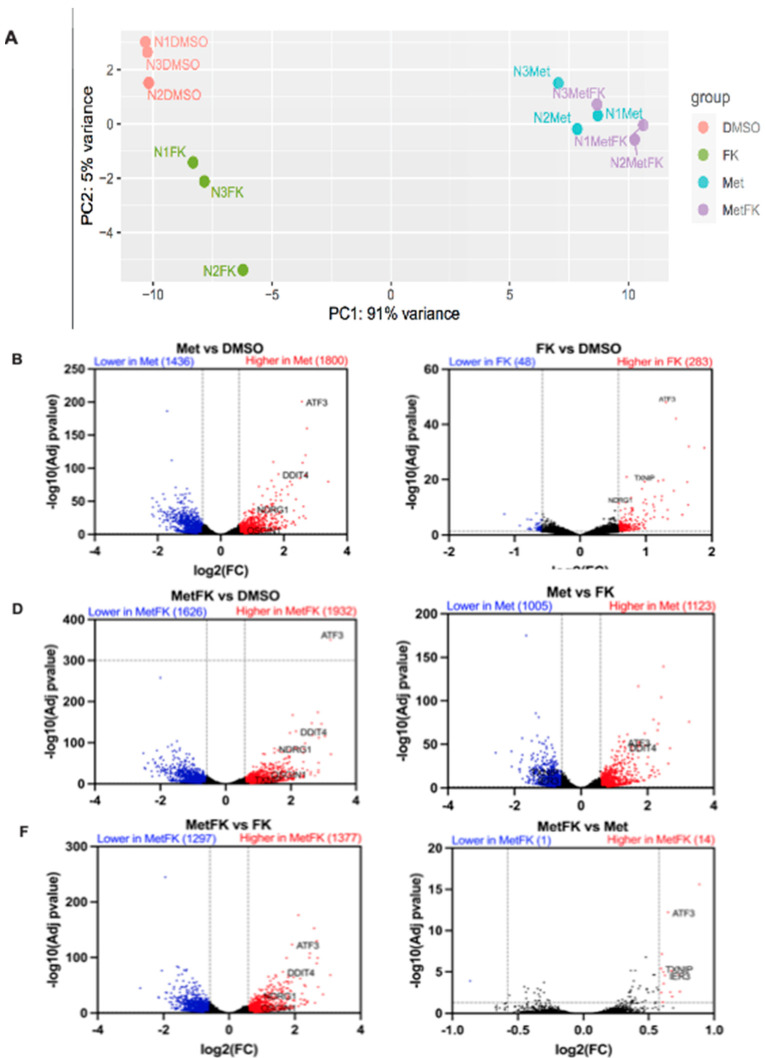
Differential gene expression in KP4 cells treated with metformin, FK866, or both. (**A**) Principal component analysis (PCA) and (**B**–**G**) volcano plots of differentially expressed genes (DEGs) from the RNAseq data obtained from KP4 cells treated as indicated for 24 h. (**A**–**G**) n = 3.

In order to obtain further insights into the mechanisms of cooperation between FK866 and metformin, we compared the transcriptomes of KP4 cells which received vehicle, metformin alone, FK866 alone, and Met+FK. Principal component analysis (PCA) showed that metformin is the main contributor to gene expression changes (91% along PC1), while FK866 only accounts for 5% of the gene expression variation along PC2 (Figure 6A). Intriguingly, while FK866-treated cells were well-separated from untreated cells along PC2, FK866 had a minimal effect in metformin-treated cells. By filtering for differentially expressed genes (DEGs) with >1.5-fold change, we found that metformin induced the expression of 1800 genes while repressing 1436 (Figure 6B). In contrast, FK866 only induced 283 genes and repressed 48 (Figure 6C). As a consequence, most of the gene expression changes caused by metformin were still detectable when compared to FK866-treated cells (1123 upregulated and 1005 downregulated genes) (Figure 6D). Similarly, when we compared Met+FK with vehicle, or FK866 alone, most of the changes reflected the effects of metformin (Figure 6E,F). However, few genes distinguished cells treated with Met+FK from those treated with metformin alone (14 upregulated genes and 1 downregulated gene) (Figure 6G).

**Figure 7:**
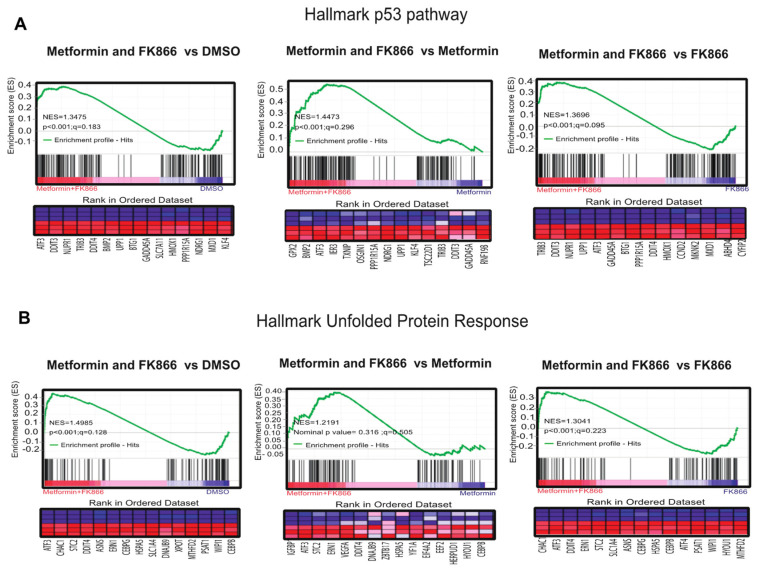

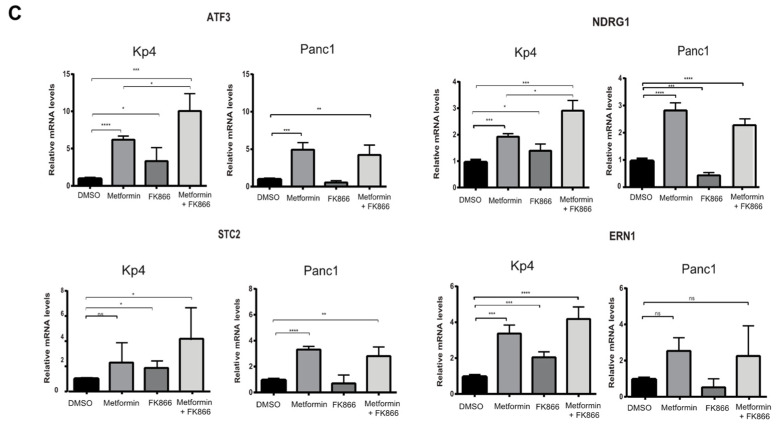
The combination of Met+FK activates the p53 pathway and the unfolded protein response (UPR). (**A**) p53 gene set that overlaps with gene expression changes in KP4 cells treated with metformin and FK866 in comparison to vehicle, metformin alone, or FK866 alone. (**B**) UPR gene set that overlaps with gene expression changes in KP4 cells treated with metformin and FK866 in comparison to vehicle, metformin alone, or FK866 alone. (**C**) RT-qPCR validation of some gene expression differences found in (**A**) and (**B**). Data are normalized over TBP and HMBS and presented as means relative to control cells (vehicle condition). Values are means of triplicates ± SEM. * *p*-value ≤ 0.05, ** *p*-value ≤ 0.01, *** *p*-value ≤ 0.001, **** *p*-value ≤ 0.0001 (ANOVA), n = 3.

In order to understand why FK866 increased metformin sensitivity, we performed GSEA, comparing cells treated with Met+FK to metformin alone, FK866 alone, and vehicle. This analysis revealed that the drug combination reactivated the p53 pathway in KP4 cells, as well as the unfolded protein response (UPR), which are both able to trigger cell death. These genes include *ATF3*, *IER3*, *OSGIN1*, *NDRG1*, *DDIT4*, *UPP1,* and *GADD34* (Figure 7A,B). The p53 target gene *ATF3* can activate up to 40% of p53 target genes, suggesting a mechanism for p53 pathway reactivation in p53 null cells [38,39,40]. IER3 plays a role in cell death induced by chemotherapy [41,42]. OSGIN1 is a mediator of apoptosis which is upregulated by oxidative stress and p53 [43]. NDRG1 is a metastasis suppressor induced by p53 and iron chelators [44,45]. TXNIP is a negative regulator of TRX, leading to oxidative stress. Intriguingly, DDIT4 (induced by metformin, p53, and p63) can form a complex with TXNIP to trigger cell death under oxidative stress [46,47]. UPP1 is a pyrimidine degrading enzyme that contributes to cancer cell death in response to drugs that induce lethal catabolic stress [48]. Finally, GADD34 is a pro-apoptotic gene induced by DNA damage, and is also regulated by ATF3 [49,50]. The UPR genes induced by the combination of Met+FK included the p53 target genes *ATF3* and *DDIT4*, as well as several chaperone and oxidative stress-related genes (*STC2*, *CEBPB,* and *ERN1*). We validated some of these genes by RT-qPCR (Figure 7C). Intriguingly, ATF3, the gene that seemed to be driving the p53 gene expression pattern in response to Met+FK, as well as the p53 target NDRG1, are induced at higher levels in KP4 cells than in PANC-1 cells. Notably, p53 is mutated in PANC-1 cells and the protein is undetectable in KP4 cells (Supplementary Figure S2), suggesting that Met+FK reactivate the p53 pathway by p53-independent effects.

## 4. Discussion

Here we provide rationale and proof-of-concept for the combined use of metformin and FK866—two compounds broadly used in preclinical studies against various types of cancer which have failed to show any beneficial effect in clinical trials [5,6,37]. We show that the combination of metformin (an OXPHOS inhibitor) with FK866 (a NAD salvage pathway inhibitor), is selectively toxic for pancreatic cancer cells. A similar combination of an OXPHOS-inhibitor with FK866 was reported to be efficient in acute myeloid leukemia (AML). In AML, the BCL2 inhibitor venetoclax targets OXPHOS, but its action is inhibited by compensatory NAD biosynthesis, which can be blocked by FK866 [51]. The mechanism of action of FK866 differs between AML blasts and stem cells. In AML stem cells, FK866 further inhibited OXPHOS, but not glycolysis. However, in AML blasts (and in ovarian and colorectal cancer cells), FK866 inhibited glycolysis [35], as we observed here in pancreatic cancer cells. This difference could be explained by tissue-specific metabolic programs that differ between AML stem cells and other cancer cells.

We found that treatment with metformin had major effects on gene expression, while FK866 had a lesser effect. In cells with combined treatment, most of the gene expression variation was due to metformin (Figure 6). This suggests that a relatively minor number of genes is linked to the higher efficacy of the drug combination. Several of these genes were associated to the p53 pathway and the unfolded protein response—both of which can lead to oxidative stress and cell death [52,53]. The UPR is often used by tumor cells as an adaptive response to chemotherapy [54], but it can become a powerful antitumor response upon prolonged activation [55]. Based on these gene expression changes, we suggest that blocking compensatory NAD biosynthesis in metformin-treated cells induces the expression of several p53 target genes and oxidative stress genes, thus leading to cancer cell death. Consistent with this idea, the NAD-dependent sirtuins SirT1 and SirT6 [56,57], as well as metformin [58], repressed *TXNIP*, one of the genes selectively increased in cells treated with Met+FK. Intriguingly, SirT1 deacetylate p53 inhibiting its activity [59], and our work suggests that NAD and sirtuins may repress some p53 target genes independently of p53. We identified ATF3 as a gene highly induced by the combination of Met+FK in KP4 cells, but less so in PANC-1 cells. NAD limitation could increase ATF3 expression by reducing sirtuin activity and increasing histone acetylation, a process that is known to regulate ATF3 expression in pancreatic cancer cells [60].

At the cellular level, the mechanism of action of the metformin-FK866 combination likely includes a cytostatic effect due to the energy crisis created by the simultaneous inhibition of OXPHOS and glycolysis, followed by cell death. The gene expression changes discussed above and the activation of the UPR could be initially adaptive, which would explain why cell death requires long term incubation with the combination, affecting a small fraction of the cells at any given time thereafter (Figure 3D). In many cancer cell lines, FK866 induces apoptosis [29]. In AML stem cells, apoptosis was secondary to lipotoxicity and oxidative stress. The lipotoxicity was likely a consequence of a reduction in the generation of monounsaturated fatty acids by the enzyme SCD that requires NAD^+^ [61]. Since the metformin–FK866 combination also induces oxidative stress, it is likely that cell death is due to the damaging effect of reactive oxygen species. Del Nagro and colleagues found that apoptosis was not the main mechanism of cell death after NAD depletion; rather, it was oncosis, a cell death pathway that includes cytoplasmic swelling and membrane disruption, ultimately leading to necrosis [62]. Consistent with this work, we found that phenformin and phenylethynylbenzyl-modified metformin did not induce apoptosis in KP4 cells in vivo, which was assessed using cleaved caspase 3 as a biomarker [63]. Two additional cell death mechanisms have been reported to be relevant to the effects of the metformin–FK866 combination. First, metformin induced the UPR and autophagy-mediated cell death in NIH-3T3 cells and in non-tumorigenic skin cells treated with phorbol esters [64]. Second, a combination of OXPHOS and glycolysis inhibitors induced parthanatos in cancer cells, a process mediated by PARP1 that affects mitochondrial activity, likely via NAD depletion [65]. Parthanatos, unlike oncosis, does not involve cell swelling, but disrupts membrane integrity, which ultimately leads to necrosis. As metformin improves the anticancer activity of NAMPT inhibitors, further studies are required in order to identify the cell death mechanism associated with the success of this combination.

### Limitations of the Study

Although the combination of metformin with FK866 was efficient in vitro for multiple cancer cell lines (Figure 2), we found that the response in vivo was different. In KP4 cells, the combination was more efficient than either drug alone, while in PANC-1 cells, the combination was no more beneficial than using FK866 alone. This could be explained by the fact that PANC-1 cells had a higher NAD^+^/NADH ratio (Figure 3) and became more resistant to metformin upon supplementation with pyruvate, α−KB, or aspartate in comparison to KP4 cells (Figure 1). These metabolites can trigger reactions that regenerate NAD^+^, mediated by enzymes such as lactate dehydrogenase [66] or malate dehydrogenase-1 [20,67]. Adding additional drugs that interfere with NAD metabolism could overcome this limitation. Notably, the NAD^+^ regenerating enzyme LDHA requires tyrosine phosphorylation to control NAD homeostasis [66], and it has recently been shown that kinase inhibitors cooperate with metformin to target a variety of cancer cells [68]. Accordingly, adding tyrosine kinase inhibitors could further improve the treatment proposed here by inhibiting NAD reoxidation. Since we detected massive necrosis in tumors treated with the combination, it is plausible that metabolites released from dying cells could have been used to compensate for NAD depletion in PANC-1 cells. In fact, extracellular NAD can enter cells, providing a pathway for dying cells to restore NAD levels in surrounding living cells, and this was previously shown to counteract FK866-induced cell death [69]. This suggests that inhibitors of NAD uptake or regeneration could be tested with the Met+FK cocktail in order to explore its anti-cancer properties in future studies. Finally, the use of immunocompromised mice may have limited the treatment, since metformin can act in part through promoting an anti-tumor immune response [70].

## 5. Conclusions

This work shows that combining metformin, a drug that inhibits complex I and reduces the NAD+/NADH ratio, with FK866, a drug that inhibits the NAD salvage enzyme NAMPT, triggers a better anticancer response than that obtained with either drug alone in several pancreatic cancer cell lines. The combination also worked in a breast cancer cell line, indicating that it can be applied to multiple tumor types.

## Data Availability

The raw data that supports this article is available upon reasonable request to G.F. RNAseq data is available from at GSE210562.

## Supplementary Materials

The following supporting information can be downloaded at: https://www.mdpi.com/article/10.3390/cancers14225597/s1, Figure S1: The combination of metformin and FK866 is not toxic in mice. Figure S2: P53 in Kp4 and Panc1 cells. Table S1: RT-qPCR primers.
