# Supplementary material for: The NAMPT Inhibitor FK866 Increases Metformin Sensitivity in Pancreatic Cancer Cells
Source: Cancers (Basel). 2022 Nov 14;14(22):5597. doi: 10.3390/cancers14225597 (PMC9688551; doi:10.3390/cancers14225597)
Supplement: Supplementary file 1 [file cancers-14-05597-s001.zip › cancers-1882249-supplementary.pdf]

cancers

MDPI

Supplementary Marterials:

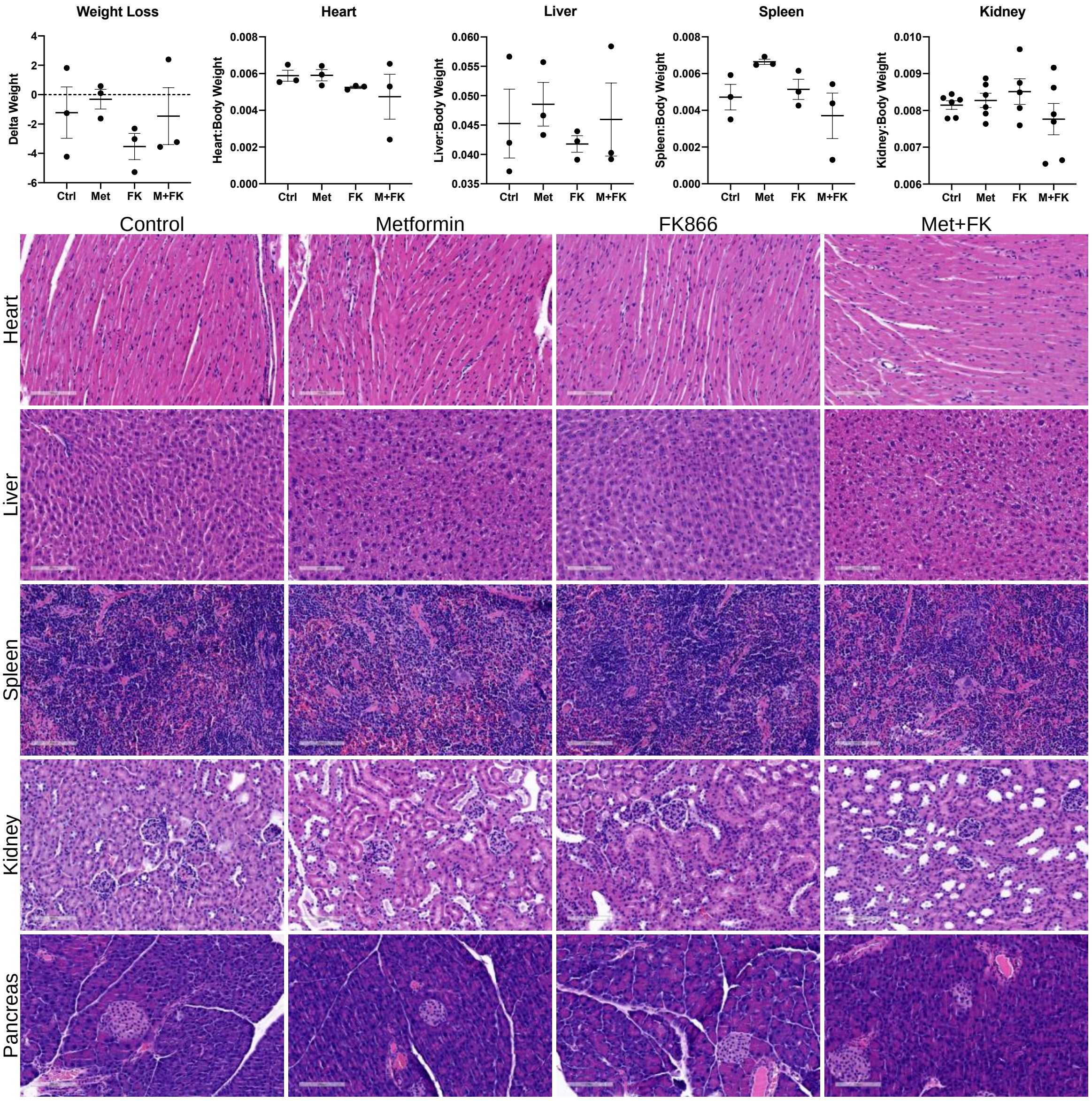

Supplementary Figure S1: The combination of metformin and FK866 is not toxic in mice. To determine if drug treatment was causing any toxicity, 3 mice per treatment group were randomly selected for necropsy once IP injections of drugs stopped after 5 weeks. Histograms show the changes in mouse weight compared to their weight on day of PANC1 subcutaneous injection and organ to weight ratios at time of necropsy. H&E staining of heart, liver, spleen, kidney, and pancreas tissues showed no histological changes between drug treatment groups in mice (n=3/group).

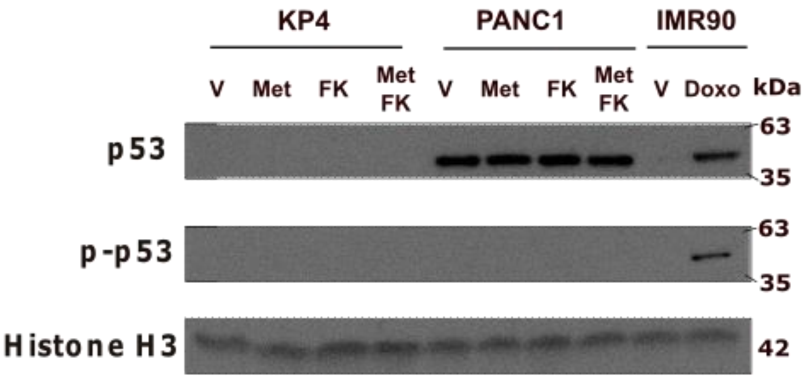

Supplementary Figure S2: P53 in Kp4 and Panc1 cells. (A) Western blot for p53 and phospho-serine 15 p53 (p-p53) in KP4 and PANC1 cells treated for 24 h in the presence or absence of 10 mM metformin (Met), 5 nM of FK866 (FK), a combination of metformin and FK866 or with vehicle (V). As a positive control, normal human fibroblasts IMR90 cells were treated with Doxorubicin (Doxo) 300ng/mL for 18h.

Table S1: RT-qPCR primers used in the study.

**Primer**	**Sequence**
qPCR primer *ATF3* Hs* forward	GGATTTTGCTAACCTGACGCC
qPCR primer *ATF3* Hs reverse	TTCCAGCTTCTCCGACTCTTTCT
qPCR primer *ERN1* Hs forward	CCTGAGGACGACGTGGACTA
qPCR primer *ERN1* Hs reverse	CATACAGAGTGGGCGTCAGC
qPCR primer *NDRG1* Hs forward	TCCTTCAACAGTTTGGGCTGA
qPCR primer *NDRG1* Hs reverse	CATCCTGAGATCTTGGAGGCG
qPCR primer *STC2* Hs forward	CCAGCACTGTTTGGTCAACG
qPCR primer *STC2* Hs reverse	TTGAGGTAGCATTCCCGCTG
†qPCR primer *HMBS* Hs forward	GGCAATGCGGCTGCAA
†qPCR primer *HMBS* Hs reverse	GGGTACCCACGCGAATCAC
†qPCR primer *TBP* Hs forward	GCTGGCCCATAGTGATCTTTGC
†qPCR primer *TBP* Hs reverse	CTTCACACGCCAAGAAACAGTGA

*Hs, Homo sapiens, † Housekeeping gene.
